# Fragility Fracture Care and Orthogeriatric Comanagement

**DOI:** 10.1155/2016/2056376

**Published:** 2016-06-27

**Authors:** Christian Kammerlander, Hitendra K. Doshi, Wolfgang Böcker, Markus Gosch

**Affiliations:** ^1^Department for General, Trauma and Reconstructive Surgery, Ludwig Maximilian University Munich, Marchioninistrasse 15, 81377 Munich, Germany; ^2^Department for Trauma Surgery and Sports Medicine, Medical University of Innsbruck, Anichstrasse 35, 6020 Innsbruck, Austria; ^3^Department of Orthopaedics and Traumatology, Tan Tock Seng Hospital, Singapore 308433; ^4^Paracelsus Medical Private University, Klinikum Nürnberg, Medizinische Klinik 2, Geriatrics, Prof.-Ernst-Nathan-Straße 1, 90419 Nürnberg, Germany

Fragility fractures are a major problem resulting in high morbidity and mortality in the older population. Over 80% of such injuries are caused by low energy trauma in patients with underlying osteoporosis. The first-year mortality rate of hip fracture ranges from 12% to 36%; only one-third of patients return to their prefracture functional status eventually and one-third require further nursing home care.

Within this special issue we have a variety of articles focussed on fragility fracture care. One article is about a new approach in treating atypical fractures. Other two articles deal with hip fractures, whereas one is showing that the routine use of cemented stems for femoral neck fracture treatment on the elderly does not lead to higher complication rates and the other article describes that the collapse following the use of a DHS for treating such fractures does impact mobility but not survival of these patients. A very interesting operative strategy is shown for proximal tibial fractures allowing the patients immediate full weight bearing after operation. One more article is focussed on the outcome of elderly hip fracture patients following prolonged ICU treatment.

In summary we have an interesting collection of valuable articles in this upcoming field. The editors want to thank the authors for their contributions.

*Christian Kammerlander* *Hitendra K. Doshi* *Wolfgang Böcker* *Markus Gosch*

